# Estimated Glomerular Filtration Rates Calculated by New and Old Equations in Children and Adolescents With Type 1 Diabetes—What to Do With the Results?

**DOI:** 10.3389/fendo.2020.00052

**Published:** 2020-02-21

**Authors:** Claudia Boettcher, Boris Utsch, Angela Galler, Corinna Grasemann, Martin Borkenstein, Christian Denzer, Bettina Heidtmann, Sascha R. Tittel, Reinhard W. Holl, DPV initiative

**Affiliations:** ^1^Paediatric Endocrinology and Diabetology, University Children's Hospital, University of Berne, Berne, Switzerland; ^2^Department of General Paediatrics and Neonatology, Centre of Child and Adolescent Medicine, Justus Liebig University Giessen, Giessen, Germany; ^3^Charité—Universitätsmedizin Berlin, Corporate Member of Freie Universität Berlin, Humboldt-Universität zu Berlin, and Berlin Institute of Health, Sozialpädiatrisches Zentrum, Berlin, Germany; ^4^Department of Paediatric Endocrinology, Klinik für Kinderheilkunde II, Universitätsmedizin Essen, Essen, Germany; ^5^Department of Paediatrics, Medical University of Graz, Graz, Austria; ^6^Division of Paediatric Endocrinology and Diabetes, Department of Paediatrics and Adolescent Medicine, University Medical Centre Ulm, Ulm, Germany; ^7^Catholic Children's Hospital Wilhelmstift, Hamburg, Germany; ^8^Institute of Epidemiology and Medical Biometry (ZIBMT), University of Ulm, Ulm, Germany; ^9^German Centre for Diabetes Research, Munich-Neuherberg, Germany

**Keywords:** diabetic kidney disease, estimated glomerular filtration rate, type 1 diabetes, children and adolescents, urinary creatinine clearance

## Abstract

**Background:** To apply and evaluate various equations for estimated glomerular filtration rates (eGFR) in a large paediatric type 1 diabetes population and compare the eGFR values with urinary creatinine clearances (UCC) in a subset of patients.

**Methods:** Six eGFR formulae applicable for children and adolescents were used for calculation of eGFR values in 36,782 children/adolescents with type 1 diabetes. Via regression models, factors influencing eGFR values were identified. eGFR values were compared with measured UCC in 549 patients. Spearman correlation coefficients were given to assess the relation of eGFR and UCC values. Bland-Altman-Plots with corresponding linear regression were drawn to evaluate the agreement between eGFR and UCC.

**Results:** eGFR values differed widely depending on the formula used, resulting in a percentage of pathological values <60 mL/min/1.73 m^2^ up to 8%. Regression models showed age, sex, and duration of diabetes as influencing factors. Microalbuminuria was associated with significantly higher eGFR values for all formulae. In comparison of eGFR with UCC, the highest correlation coefficient was 0.33, the lowest 0.01. Bland-Altman-Plots demonstrated graphically a poor agreement between eGFR and UCC, regardless of the formula used.

**Conclusions:** The broad range of eGFR values indicate that an ideal eGFR formula for children and adolescence with T1D is yet missing. The minimal agreement between measured UCC and eGFR values urges us to be careful in application and interpretation of eGFR values regardless of the formula used.

## Introduction

Diabetic nephropathy remains a common complication in patients with diabetes; the prevalence of diabetic nephropathy in individuals with diabetes younger than 18 years of age is estimated to be 3.44% in the United States (1). Diabetes is the main cause of end-stage renal disease (ESRD), with type 1 patients facing a 30-year cumulative incidence for ESRD of 3.3–7.8% (2). Identifying and monitoring diabetic kidney disease requires *inter alia* assessment of kidney function. This can be done by determining the glomerular filtration rate (GFR). As the measurement of GFR using the clearance of exogenous substances such as inulin is cumbersome, serum creatinine concentration combined with other easily determined parameters (height, weight, sex, etc.) is most frequently used for estimating GFR (eGFR) and is recommended by the American Diabetes Association and International Society of Nephrology (3, 4). In paediatrics still in use as a screening method for renal disease are timed urinary creatinine clearances—despite the known main limitation of incomplete urine collections—as it is a non-invasive tool.

Many eGFR-equations that are in use today were originally designed for adults and therefore less suitable for children, as proven in numerous studies (5–8). However, over the last 15 years a whole string of equations (based on serum creatinine) more appropriate or even designed for children were published (9–12). In this study, we aimed to give an overview of GFR estimated by various newer and older formulae in children and adolescents with type 1 diabetes (T1D), to identify influencing factors and to compare eGFR values obtained with different formulae with creatinine clearance based on timed urine collections (UCC).

## Methods

### Data Source

The German-Austrian-Swiss-Luxembourgish DPV (Diabetes-Patienten-Verlaufsdokumentation) Initiative database comprises prospective data of 485 diabetes centres. Demographic, anthropometric, and diabetes-related characteristics are documented for quality assurance and scientific research. The institutional review board of Ulm University and the local institutional review boards approved analysis of anonymised DPV data.

### Study Population

A total number of 36,782 patients with T1D out of 365 paediatric diabetes centres were identified in the DPV database according to the following criteria: age 1–<18 years, serum creatinine value, height and weight data available, and time period 2010–2018.

### Variables

Demographic variables of our study group included age, sex, and duration of diabetes. The variable age was categorised as 1–<6, 6–<12, and 12–<18 years. Height and weight were used to calculate the BMI (weight [kg]/height [m^2^]) and the respective BMI-SD scores (categorised as <90th percentile, ≥90–97th percentile, and ≥97th percentile) as a measure of overweight and obesity according to recent reference data (13). Systolic and diastolic blood pressure resulted in a diagnosis of “hypertension” (>95th percentile of age specific reference values) (14). Antihypertensive medication was documented. Glycaemic control was assessed by HbA_1c_ levels. In order to equalise for different laboratory methods, HbA_1c_ data were mathematically standardised to the DCCT reference range (4.05–6.05% [20.8–42.6 mmol/mol]) (15) and classified into three categories: HbA1_c_ <7.5% [<58.5 mmol/mol], 7.5–<9.0% [58.5–74.9 mmol/mol], and ≥ 9.0% [≥74.9 mmol/mol]. IDMS traceable serum creatinine values served for calculation of eGFR, using different GFR estimation formulae: four formulae developed especially for use in children (IDMS-traceable formula by Schwartz [eGFR_Schwartz_short], Schwartz-Lyon-formula [eGFR_SchL], Full-age-spectrum with Q-age extension [eGFR_FAS_QA], Full-age-specrum with Q-height extension [eGFR_FAS_QH], as well as two formulae originally designed for adults (Lund-Malmö [eGFR_LM], Lund-Malmö with lean body mass [eGFR_LM_LBM]) (Table 1). When available, a measured urinary Creatinine Clearance (UCC)—via timed urine collection and concomitant blood sample—was documented. UCC –values below 50 mL/min/1.73 m^2^ were excluded. Microalbuminuria, defined as at least two positive urine albumin tests out of three consecutive tests (random spot collection) and diabetic retinopathy (ophthalmological diagnosis) were registered. For each patient, data of the most recent year of follow-up were analysed.

**Table 1:** eGFR formulae used in this study.

**Name**	**Formula**
IDMS-traceable “Short” Schwartz (eGFR_Schwartz_Short) (9)	= 41.3 * (*height [m]/serum creatinine* $\left[\frac{mg}{dL}\right]$
Schwartz-Lyon (eGFR_SchL) (10)	= k* (*height* [*m*]/*serum creatinine* $\left[\frac{mg}{dL}\right]$ with *k* = 41.3 for boys >13 years of age and *k* = 36.7 for all others
Full-age-spectrum with Q-age extension (eGFR_FAS_QA) (11)	= $107.3/serum\;creatinine\;\left[\frac{mg}{dl}\right]/Q\;\left[\frac{mg}{dL}\right]$ with *Q* = 0.21+0.057 * age (*years*)−0.0075 * *age*^2^+0.00064 * *age*^3^−0.000016 * *age*^4^ for boys with *Q* = 0.23+0.034 * *age* (*years*)−0.0018 * *age*^2^+0.00017 * *age*^3^−0.0000051 * *age*^4^ for girls
Full-age-spectrum with Q-height extension (eGFR_FAS_QH) (11)	= $107.3/serumcreatinine\;\left[\frac{mg}{dl}\right]/Q\;\left[\frac{mg}{dL}\right]\;$ with *Q* = 3.94−13.4 * *height* [*m*]+17.6 * *height*^2^−9.84 * *height*^3^+2.04 * *height*^4^
Lund-Malmö (eGFR_LM) (12)	= *e*^*Z*−0.0124* *age* [*years*]+0.339* ln(*age*)^ if male = *e*^*Z*−0.0124* *age* [*years*]+0.339* ln(*age*)−0.226^ if female with e = *the base of the natural logarithm* (*ln*) *and* *Z* = 4.62−0.00112 * $serum\;creatinine\;\left[\frac{mg}{dL}\right]$ if serum creatinine <150 μmol/L *Z* = 8.17+0.0005 * *serum creatinine*−1.07 * in (*serum creatinine*) $\left[\frac{mg}{dL}\right]$) if serum creatinine ≥ 150 μmol/L
Lund-Malmö with lean body mass (eGFR_LM_LBM) (12)	= *e*^*Z*−0.00587* *age* [*years*]+0.00977* *LBM*^ with e = the base of the natural logarithm (ln) and *Z* = 4.95−0.0110 * $serum\;creatinine\;\left[\frac{mg}{dL}\right]$ if serum creatinine < 150 μmol/L *Z* = 8.58+0.0005 * *serum creatinine*−1.08 * (*serum creatinine*) $\left[\frac{mg}{dL}\right]$) if serum creatinine ≥ 150 μmol/L with lean body mass (LBM) = 1.07 * *weight* [*kilogramm*]−148 * (*weight*/*height* [*m*])^2^
Measured creatinine Clearance (16) (UCC)	= (*urinary creatinine* $\left[\frac{mg}{dL}\right]$ * *urine volume* [*ml*] /(*serum creatinine* $\left[\frac{mg}{dL}\right]$ * *time* [min]) * 1.73/*BSA*

### Statistical Analysis

For descriptive analysis, mean, SD (continuous variables), median with lower and upper quartile and percentages (categorical variables) were provided. Kruskal-Wallis-tests (continuous variables) and χ^2^-tests (binary variables) were applied for group comparisons. *P* values were adjusted according to Holm (Bonferroni-stepdown) for multiple comparisons. To investigate the effect of potentially influencing factors on the eGFR, linear regression models were used. *F* tests were deployed to test for differences between groups; adjusted means (least squared means) were calculated based on observed marginal frequencies. Spearman correlation was used to assess the relation of eGFR values obtained with different formulae and UCC values. Bland-Altman-Plots with corresponding linear regression were utilized to evaluate the agreement between eGFR and UCC. All analyses were performed with the SAS for Windows version 9.4 software (SAS Institute, Cary, NC, USA).

## Results

### Characterisation of Study Cohort

Of a total of 54,393 children and adolescents aged 1–<18 years with T1D followed in the DPV database, 36,782 had a documented serum creatinine value and complete auxological data, of whom 549 patients with a 24-h urine /blood sample for the calculation of UCC. Of the final study population, 46.6% (*n* = 17,146) were female. Mean age was 14.0 ± 3.8, median age 15.4 [11.7 lower Quartile-−17.3 upper Quartile] years with 5.0% of the patients <6 years (mean 4.2 ± 1.3, median 4.4 [3.2–5.3] years), 21.4% between 6 and <12 years (mean 9.5 ± 1.7, median 9.7 [8.1–10.9] years), and 73.6% >12 years of age (mean 16.0 ±1.7, median 16.8 [14.8–17.4] years). Average duration of diabetes was 5.5 ± 4.2 years. Overall, 40.8% of the children/adolescents had an HbA1_c_ value <7.5, 36.8% between 7.5 and 9%, and 22.4 ≥ 9%. Mean serum creatinine was 0.42 ± 0.15 mg/dl in the age group <6 years, 0.55 ± 0.15 mg/dl in the age group 6–<12 years and 0.74 ± 0.18 mg/dl in the age group 12–18 years; mean BMI-SDS was 0.28 ± 0.94. Of the population that was screened for diabetic complications, 0.5% showed diabetic retinopathy, 7.0% microalbuminuria (age group <6 years 2.7%, age group 6–<12 years 5.2%, 12–<18 years 7.7%), 20.7% fulfilled the criteria for “hypertension” and 2.6% took antihypertensive medication.

### GFR Estimated via Different Formulae and Influencing Factors

Table 2 gives an overview of eGFR, calculated by six different formulae, and UCC. Presented are mean eGFR values for the whole population and for subgroups according to sex, age, and BMI. The percentages of eGFR-values <60 ml/min/1.73 m^2^ are included. Mean eGFR ranged between 95.3 ± 16.0 mL/min/1.73 m^2^ (eGFR_LM) and 110.4 ± 26.0 mL/min/1.73 m^2^ (eGFR_FAS_QH) in all patients, with the results of the Schwartz_Short-formula being in between the two extremes (102.8 ± 24.1 mL/min/1.73 m^2^). For females, the lowest [highest] mean eGFR was seen when calculated by the eGFR_LM-Formula (88.1 ± 11.9 mL/min/1.73 m^2^) [eGFR_FAS_QH (109.6 ± 25.3 mL/min/1.73 m^2^)]; for males, the calculation via SchwL-formula showed the lowest (95.3 ± 21.1 mL/min/1.73 m^2^), and the calculation via FAS-QH the highest (111.1 ± 26.6) mean eGRF. In the age group 1–<6 years mean eGFR varied between minimal 93.4 ± 17.6 (eGFR_LM) and 114.2 ± 33.1 mL/min/1.73 m^2^ (eGFR_Schwartz_Short), in the age group 6–<12 between 98.6 ± 22.0 (eGFR_SchwL) and 111.0 ± 24.7 mL/min/1.73 m^2^ (eGFR Schwartz_short), and in the age group 12–<18 between 93.6 ± 15.2 (eGFR_LM) and 112.2 ± 26.1 mL/min/1.73 m^2^ (eGFR_FAS_QH). Regarding the BMI-subgroups, the BMI group ≥ 97 P showed the highest variation for mean eGFR: 94.6 ± 15.5 (eGFR_LM) −116.1 ± 17.2 mL/min/1.73 m^2^ (eGFR_LM_LBM).

**Table 2:** eGFR values (ml/min/1.73 m^2^) and UCC in all patients and subgroups according to gender, age, and BMI as well as percentages of eGFR values < 60 ml/min/1.73 m^2^; unadjusted comparison female to male group: ***p < 0.0001; unadjusted comparison age group 1–<6 and age group 6–<12: ^
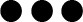
^*p* < 0.0001, ^
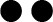
^*p* < 0.001, ^
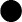
^*p* < 0.05; unadjusted comparison age group 6–<12 and age group 12–<18: ^
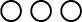
^*p* < 0.0001; unadjusted comparison age group 1–<6 and age group 12–<18: ^
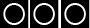
^*p* < 0.0001; unadjusted comparison BMI group <P90 and BMI group ≥ P90–97: ^
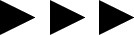
^*p* < 0.0001; unadjusted comparison BMI group ≥ P90–97 and BMI group ≥ P97: ^
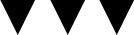
^*p* < 0.0001, ^
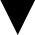
^*p* < 0.05; unadjusted comparison BMI group < P90 and BMI group ≥ P97: ^
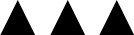
^*p* < 0.0001; *P*, Percentile.

	***N***	**eGFR_Schwartz_short**		**eGFR_SchL**		**eGFR_FAS_QA**		**eGFR_FAS_QH**		**eGFR_LM**		**eGFR_LM_LBM**		**UCC**	
		**Mean** **± SD**	**% <60**	**Mean** **± SD**	**% <60**	**Mean** **±SD**	**% <60**	**Mean** **± SD**	**% <60**	**Mean** **±SD**	**% <60**	**Mean** **±SD**	**% <60**	***N***	**Mean** **±SD**
**All patients**	36,782	102.8 ± 24.1	1.7	95.2 ± 21.3	2.7	108.2 ± 23.9	1.5	110.4 ± 26.0	1.4	95.3 ± 16.0	1.4	105.0 ± 15.0	0.7	549	122.3 ± 35.6
**Sex**															
**Female**	17,146	*** 107.0 ± 24.2	1.4	95.1 ± 21.5	2.9	*** 108.9 ± 24.4	1.6	*** 109.6 ±25.3	1.6	*** 88.1 ±11.9	1.9	104.9 ± 14.1	0.6	263	123.1 ±31.7
**Male**	19,636	99.2 ± 23.3	1.9	95.3 ± 21.1	2.5	107.6 ± 23.5	1.4	111.1 ± 26.6	1.3	101.7 ± 16.4	1.0	105.1 ± 15.6	0.7	286	121.6 ±38.9
**Age (yrs)**															
**1–<6**	1,849	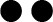 114.2 ± 33.1	4.6	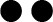 101.5 ± 29.5	7.6	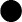 100.5 ± 29.3	8.1	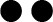 103.0 ± 30.0	6.9	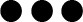 93.4 ± 17.6	3.6	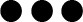 108.3 ± 13.6	0.5	27	144.5 ±56.8
**6–<12**	7,879	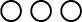 111.0 ± 24.7	1.7	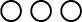 98.6 ± 22.0	3.4	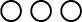 102.9 ± 23.2	2.7	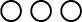 106.0 ± 23.8	2.3	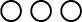 101.7 ± 16.7	1.2	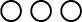 105.8 ± 13.4	0.7	92	125.1 ± 36.3
**12–<18**	27,054	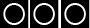 99.7 ± 22.3	1.4	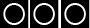 93.8 ± 20.2	2.1	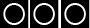 110.2 ± 23.4	0.7	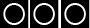 112.2 ± 26.1	0.8	93.6 ± 15.2	1.3	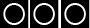 104.6 ± 15.4	0.7	430	120.3 ± 33.3
**BMI**															
** < P 90**	31,553	102.7 ± 24.0	1.7	95.3 ± 21.3	2.7	108.1 ± 24.0	1.5	110.4 ± 26.0	1.5	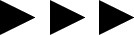 95.6 ± 16.1	1.4	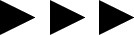 103.8 ± 14.4	0.7	481	121.9 ± 35.3
**≥P90–97**	3,881	103.2 ± 23.9	1.3	94.8 ± 21.0	2.3	108.0 ± 23.7	1.3	110.1 ± 25.8	1.0	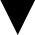 93.5 ± 15.3	1.4	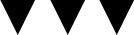 111.0 ± 15.4	0.4	51	126.7 ± 39.2
**≥P97**	1,346	103.9 ± 24.7	1.6	96.0 ± 21.7	2.7	109.2 ± 25.1	1.9	111.3 ± 26.3	1.6	94.6 ± 15.5	1.6	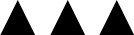 116.1 ± 17.2	0.5	17	120.8 ± 34.3

**Figure 1:**
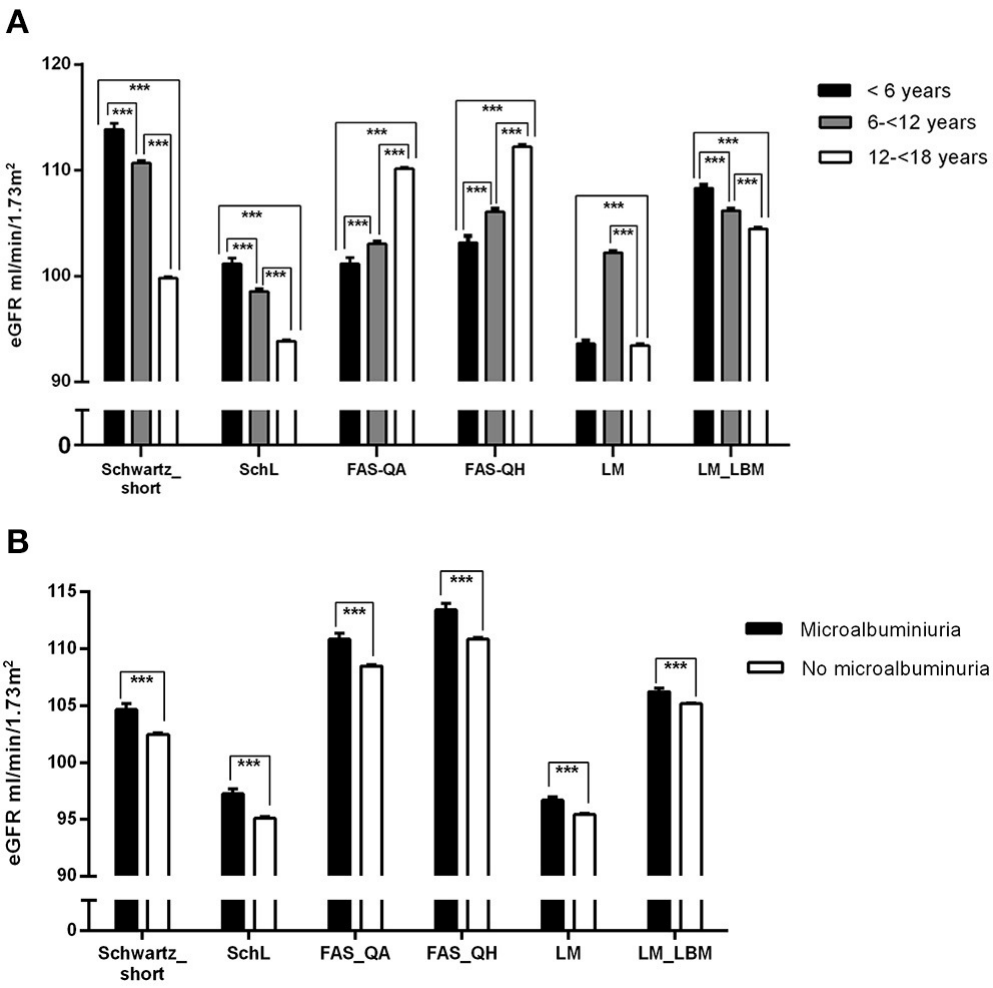
**(A)** eGFR (36,780 patients) calculated by different formulae for three age groups. Estimates (± standard error) are derived from linear regression models adjusted for age, sex, diabetes duration, and BMI. ***p < 0.0001. **(B)** eGFR (27,744 patients) sorted into two groups by the characteristic “microalbuminuria” or “no microalbuminuria,” respectively. Estimates (± standard error) are derived from linear regression models, adjusted for age, sex, diabetes duration, BMI, HbA1c, antihypertensive medication. ***p < 0.0001.

Linear regression analyses adjusted for age, sex, duration of diabetes and BMI, identified age for all formulae (Figure 1A) and sex for all but the Schwartz-Lyon formula as significant influencing factors. Diabetes duration seemed to affect Schwartz-short-, Schwartz-Lyon-, FAS-QA-, Lund-Malmö-, and Lund-Malmö-LBM-formulae. Introducing microalbuminuria or diabetic retinopathy into the regression models (adjusted for age, sex, duration of diabetes, BMI, HbA1c, antihypertensive drugs) revealed a significant association between microalbuminuria and eGFR according to all six formulae (increased eGFR-values when microalbuminuria present) (Figure 1B), whereas a diagnosis did not take effect in any formula.

### eGFR in Correlation With UCC and With Each Other

**Figure 2:**
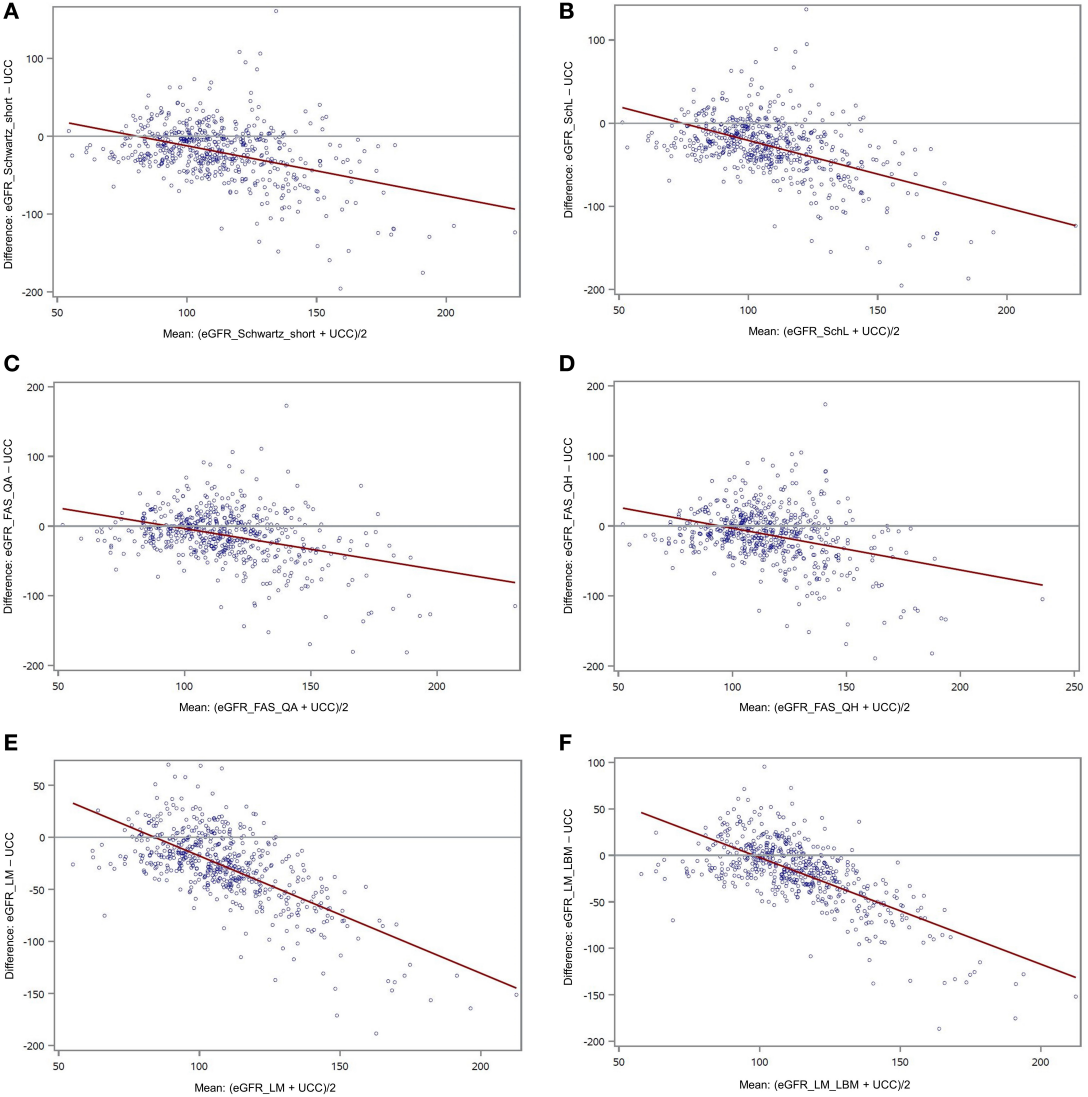
**(A–F)** Bland-Altman-Plots (plot of differences) between eGFR (**A**: eGFR_Schwartz_Short; **B**: eGFR_Schw_L; **C**: eGFR_FAS_QA; **D**: eGFR_FAS_QH; **E**: eGFR_LM; **F**: eGFR_LM_LBM) and UCC with red regression line.

Using the Spearman correlation, Table 3 shows the relation of eGFR (all formulae) and UCC. The maximal correlation coefficient was 0.30 for eGFR_Schwartz_Short (all patients) and 0.33 also for eGFR_Schwartz_Short for the age group 12–<18 years. Figures 2A–F presents Bland-Altman-Plots (difference vs. average) with regression line between UCC and all six formulae.

**Table 3:** Spearman correlation coefficients for eGFR and UCC, all patients, and age groups.

	**UCC** ***N* = 549**	**UCC age <6 yrs** ***N* = 27**	**UCC age 6–<12 yrs** ***N* = 92**	**UCC age 12–<18 yrs** ***N* = 271**
eGFR_Schwartz_short	0.30^***^	0.11	0.14	0.33^***^
eGFR_SchL	0.29^***^	0.11	0.14	0.32^***^
eGFR_FAS_QA	0.27^***^	0.09	0.19	0.32^***^
eGFR_FAS_QH	0.20^***^	0.09	0.14	0.24^***^
eGFR_LM	0.25^***^	0.01	0.16	0.28^***^
eGFR_LM_LBM	0.26^***^	0.11	0.10	0.29^***^

*** *p < 0.0001*.

## Discussion

In our large paediatric population, the application of various formulae for GFR estimation results in pretty far-flung values (see Table 2). A similar or even greater variability is visible when considering subgroups, e.g., sorted by age or BMI. These results and the fact that so many formulae have been developed in the first place visualises that the “ideal” eGFR-formula based on serum creatinine might not exist. The Schwartz-short formula was evaluated in children *with chronic kidney disease* (9). Indeed, this formula was found to be reliable and accurate in children and adolescents and in young adults with mild to moderate kidney impairment (5), but not necessarily in other patient groups or healthy children (17, 18). Hence, compared to median GFRs of a meta-analysis looking into the measured GFR of children and adolescents, and claiming that the data could be considered as reference data (19), the eGFRs via Schwartz-short of our cohort showed similar levels. The Schwartz-Lyon formula is the further development of the Schwartz-short formula with the intention to improve the performance by introducing two k-coefficients. Due to the *k*-coefficient of 36.7 in the Schwartz-Lyon formula (Schwartz-short: *k* = 41.3) for all patients but the males >13 years of age (*k* = 41.3), we saw one of the lowest mean eGFR-levels (generally and in the subgroups) of the study, and lower levels compared to the levels of the meta-analysis-study mentioned above (19). In 2012, the group around Pottel et al. published “A simple height-independent equation for estimating GFR in children” that was valid for Children aged <14 years (20). Quickly, this formula was refined by the FAS_QA- and FAS_QH-formulae which pursued the goal to provide equations for all ages, especially without the problematic discontinuity between paediatric and adult equations (21). The principle behind those new formulae were age- and sex-specific (median) serum creatinine values, or height dependent values, respectively, on the basis of a large Belgian data set of *healthy* subjects aged 0.1–20 years. Applying these two formulae to our cohort resulted in a shift towards higher eGRF-values, generally and in almost all subgroups with eGFR_FAS_QH > eGFR_FAS_QA-values. The increase was especially accentuated for the male adolescents, reflecting the effort to create formulae that meet the challenges of non-linear serum creatinine rise above 14 years of age, particularly in boys, and the large growth differences. The Lund-Malmö (LM)- and Lund-Malmö-Lean-Body-mass (LM_LBM)-formulae were originally designed for adults, and developed on the basis of patients mostly with renal disease (12). In a second step, the formulae were evaluated in a small group of children, many of them again with suspected or confirmed renal disease (22). The eGFR-values produced by the LM-formula, that takes age and—via a constant factor—sex into account, were among the lowest in our population. Compared to the meta-analysis data of Pottel (19), we can assume an underestimation of GFR. The introduction of the factor lean body mass by using the LM-LBM-formula resulted in higher values with the highest values in the group BMI ≥ P97—reflecting the newly included parameters weight and height.

At a GFR threshold of <60 mL/min/1.73 m^2^, approximately 50% of an adult's normal kidney function is lost. This cut-off is widely used for the definition of chronic kidney disease in adults and in children aged > 2 years and adolescents (23), although there is data that a threshold of <75 mL/min/1.73 m^2^ could be more accurate in children (24). The range of eGFR-values considered to be definitively pathological (<60 mL/min/1.73 m^2^) varied between 0.4% (eGFR_LM_LBM, subgroup BMI ≥P90–97) and 8.0% (eGFR_FAS_QA, subgroup age 1–<6 years) in our study, with the age group 1–<6 years showing the highest percentages of pathological eGFRs over all formulae. We would expect the youngest of the patients with type 1 diabetes (with the lowest mean duration of diabetes) to be those with the least pathological values. A Belgian 2015-study used the FAS-QA-formula in 8,505 subjects aged 2–25 years who were registered in a large hospital database and assumedly in good renal health. This study reported eGFR-values <60 mL/min/1.73 m^2^ in the age group 2–6 with a maximal frequency of 0.65% (24). The FAS-QA-formula is height independent, that leaves two possibilities to explain the discrepancy (and the clear GFR underestimation): either a variation in the serum creatinine values. This seems very unlikely as all our study centres employed IDMS-traceable creatinine measurements, as the dataset originally used for the FAS-QA-formula. Alternatively, the Q-values (median of serum creatinine of the Belgian population the formula was developed in) might not be suitable for our German-Austrian-Swiss-Luxembourgish population in that age group. Generally spoken, we have to be aware, that differences among formulae using the same filtration markers (here: creatinine) mirror differences in the variables included in the equations and the forms and coefficients of the variables. The variables are chosen and adapted to the population the formulae were developed and elaborated in. We have to think carefully whether e.g., Schwartz-Short-formula (developed in a chronic-kidney disease population) or FAS-QH-formula (developed in healthy European children and adolescents) in which age group might be more suitable to estimate GFR in a population of children and adolescents with type 1 diabetes—alternatives to the “classical” paediatric Schwartz-short-formula are available.

What factors could be identified as influencing our eGFR-values in regression analyses? Age and sex were significant influencing factors for all (age) or almost all (sex) eGFR values, but not always in the same direction: Schwartz-short, Schwartz-Lyon, and Lund-Malmö-LBM formulae showed the younger the higher eGFR-values whereas the “FAS-family” exhibited the older the higher eGFR-values. Using Schwartz-short- and FAS-QA-Formula meant higher eGFR-values in females than in males. For FAS-QH, Lund-Malmö- and Lund-Malmö-LBM-formulae the contrary was true. It is recognized that between the age of 1 and 2 years (measured) GFR (scaled for Body Surface Area BSA) reaches adult levels (18, 25, 26). However, serum creatinine levels that serve as a basis for estimating GFR change during growth mainly due to accretion of muscle, especially in boys, until adolescence (27). Based on the assumption of a more close linkage of muscle mass and height as opposed to weight or BSA (28), the Schwartz-short- and Schwartz-Lyon-formula introduced height into the equation. As the original population for the Schwartz-formula were children with chronic kidney disease with possible short stature, this formula possibly underestimates GFR in adolescence. Schwartz-Lyon tries to compensate for the factor (male) sex via a different *k*-coefficient for boys. The “FAS-family”-approach consisted of the use of population normalized (age related) Q-values, based on median serum creatinine values. In a further step the FAS-formula was modified by including age (FAS-QA) or height (FAS-QH) and gender in a sophisticated way—but still leaves us with age as an influencing factor as shown in our results. For the factor sex, we have to keep in mind, that despite all attempts to correct for sex in direct (Schwartz-Lyon-, FAS-QA-, Lund-Malmö-formula) or indirect (Schwartz-short-, FAS-QH, Lund-Malmö-LBM-formula) ways, we still can see an influence on the eGFR-values for most formulae.

Diabetes duration seemed to be another influencing factor for five formulae except FAS-QH-formula. The differences in absolute numbers though between the group “diabetes duration <2 years” compared to “diabetes duration ≥ 2 years” were marginal, and the direction inconsistent so that this significance might be only due to large numbers.

Albuminuria is still seen as a biomarker of renal damage in patients with diabetes, although no longer necessarily believed to be the initial manifestation of chronic kidney disease (29). The percentage of patients with microalbuminuria in our study was comparable to other studies in paediatric T1D populations (30, 31). Regression models showed a significant association between microalbuminuria and eGFR-value in all formulae: patients with microalbuminuria had significantly higher eGFR values than those without microalbuminuria. Literature tells us, that a high (measured) GFR in the means of glomerular hyperfiltration may precede microalbuminuria (30, 32, 33) as a sign of diabetic nephropathy, and that a high eGFR is associated with a more rapid loss of kidney function (34). However, the link between hyperfiltration and subsequent albuminuria or eGFR loss in humans has not been consistently confirmed (35). A study of Perrin et al. even stated, that eGFR cannot accurately replace measured GFR to detect hyperfiltration (36). Nevertheless, we think that our findings are indicative for a possible renal impairment. Patients with increasing eGFR and microalbuminuria should be carefully monitored.

In clinical practice, GFR in children is often measured by creatinine clearance, relating serum creatinine levels to timed urinary creatinine excretion. This method is substantially less invasive than e.g., inulin clearance and therefore still in use although it is not the most accurate method and overestimates inulin clearance (37). One of the method's limitations is incomplete urine collection. The urine collection of the 549 children include in our study took mainly place during hospitalisations, for example in the course of initiation pump therapy. Therefore, we can assume accurate and complete collections under supervision of trained personnel. Another limit particularly for our study is the small number of patients with UCC compared to the number of patients where eGFR could be calculated. But the UCC-population could be considered as a representative sample of the total study-population as there were no significant differences concerning age, diabetes duration, HbA1c, BMI, serum-creatinine or retinopathy/microalbuminuria rate. For all patients, the eGFR_Schwartz_short-formula showed the highest correlation (correlation coefficient 0.30) between eGFR-values and UCC the lowest (0.20) the eGFR_FAS_QH-formula. Although there is a positive correlation between UCC and eGFR-values determined with different formulae, correlation is not good at all. Obviously UCC and our “paediatric” eGFR-values are two different things, despite the fact that both methods are based on creatinine. This is in contrast to a large study in young adult T1D patients that investigated the correlation between UCC and eGFR, but used “adult” formulae (38).

The Bland-Altman-Plots describe the agreement between UCC- and eGFR-values (difference against average). Ideally, all the differences would be equal to zero, and the regression line would be horizontal (39). This is not the case in our study. In all six formulae, the differences that equal zero are scarce, and clustered around “normal” values (75–110 ml/min/1.73 m^2^); the higher the average, the higher the negative difference, resulting in a skewed regression line. We interpret this finding that eGFR is rather suitable for detecting low eGFR values and less for higher values as the case may be in the state of hyperfiltration (>130 ml/min/1.73 m^2^). Hyperfiltration is a common phenomenon in T1D, especially in early diabetes and as a possible sign of (future) renal damage (40). A Swedish study in children and young adults with T1D showed similar results: they measured GFR by inulin clearance, and calculated eGFR using different formulae, but had to state that eGFR does not reliably detect hyperfiltration (36).

Unfortunately non-creatinine endogenous biomarker values (e.g., cystatin c, β-trace protein or β2-microglobulin) as an alternative to the urinary creatinine clearance method were not available for our population. The same is true for data on clearances with exogenous filtration markers, which are considered as gold standard in the assessment of renal clearance function. Such data would have been ideal for comparison with the GFR-values estimated by various formulae. The lack of those can be considered as a true limitation. But due to the “real-life” conditions of the study we were fortunate to have over 500 urinary creatinine clearances, keeping in mind that the purpose of those was screening children/adolescents with type 1 diabetes for renal disease.

## Conclusion

Our study showed that application of different formulae for estimating GFR in a large population of T1D children and adolescents leads to a broad range of eGFR values. The population each formula was developed for has to be kept in mind. None of the formulae seems to be perfect over all subgroups we analysed. Despite attempts to take into account e.g., age and sex and integrate those factors in a number of ways, age and sex are anyhow recognised as influencing factors on eGFR in regression analyses. Correlations between measured UCC and eGFR were poor, and Bland-Altman-plots revealed poor agreement between the two methods, especially for eGFR values in the hyperfiltration range. We conclude, that eGRF-calculations in children and adolescents with T1D have to be regarded with extremely cautious attitude and—if in doubt—have to be replaced by measured GFR, for example inulin clearance.

## Data Availability Statement

The raw data supporting the conclusions of this article will be made available by the authors, without undue reservation, to any qualified researcher.

## Ethics Statement

The studies involving human participants were reviewed and approved by Institutional review board of Ulm University. Written informed consent to participate in this study was provided by the participants' legal guardian/next of kin.

## Author Contributions

CB, ST, and RH contributed substantially to the conception and design of the work as well as to the analysis and interpretation of data for the work. CB drafted the manuscript. All authors are responsible for data acquisition for the work, revised it critically for important intellectual content, provided approval for publication of the content, and agree to be accountable for all aspects of the work in ensuring that questions related to the accuracy or integrity of any part of the work are appropriately investigated and resolved.

### Conflict of Interest

The authors declare that the research was conducted in the absence of any commercial or financial relationships that could be construed as a potential conflict of interest.
