# Supplementary material for: Estimated Glomerular Filtration Rates Calculated by New and Old Equations in Children and Adolescents With Type 1 Diabetes—What to Do With the Results?
Source: Front Endocrinol (Lausanne). 2020 Feb 21;11:52. doi: 10.3389/fendo.2020.00052 (PMC7046626; doi:10.3389/fendo.2020.00052)
Supplement: Supplementary file 1 [file Table_1.DOCX]

Supplementary Material

# Supplementary Data

The following paediatric diabetes centres contributed to the work:

Aachen - Uni-Kinderklinik RWTH, Aalen Kinderklinik, Ahlen St. Franziskus Kinderklinik, Altötting-Burghausen Innere Medizin, Amberg Kinderklinik St. Marien, Arnsberg-Hüsten Karolinenhosp. Kinderabteilung, Aue Helios Kinderklink, Augsburg IV. Med. Klinik, Augsburg Josefinum Kinderklinik, Augsburg Kinderklinik Zentralklinikum, Aurich Kinderklinik, Bad Aibling Internist. Praxis, Bad Hersfeld Kinderklinik, Bad Kreuznach-Viktoriastift, Bad Kösen Median Kinderklinik, Bad Mergentheim - Diabetesfachklinik, Bad Mergentheim - Kinderdiabetologische Praxis, Bad Oeynhausen Herz-und Diabeteszentrum NRW, Bad Orb Spessart Klinik, Bad Orb Spessart Klinik Reha, Bad Reichenhall Kreisklinik Innere Med., Bad Salzungen Kinderklinik, Bad Säckingen Hochrheinklinik Innere, Bad Waldsee Kinderarztpraxis, Basel Uni-Kinderspital beider Basel (UKBB), Bautzen Oberlausitz KK, Bayreuth Innere Medizin, Berchtesgaden CJD, Berchtesgaden CJD-Beruf.REHA, Berlin DRK-Kliniken Pädiatrie, Berlin Endokrinologikum, Berlin Klinik St. Hedwig Innere, Berlin Lichtenberg - Kinderklinik, Berlin Parkklinik Weissensee, Berlin Virchow-Kinderklinik, Berlin Vivantes Hellersdorf Innere, Bern Universitätsklinik für Diabetologie und Endokrinologie, Bielefeld Kinderarztpraxis, Bielefeld Kinderklinik Gilead, Bocholt Kinderklinik, Bochum Universitäts St. Josef, Bochum Universitätskinderklinik St. Josef, Bonn Uni-Kinderklinik, Bottrop Knappschaftskrankenhaus Innere, Braunschweig Kinderarztpraxis, Bremen - Kinderklinik Nord, Bremen - Mitte Innere, Bremen Zentralkrankenhaus Kinderklinik, Bremerhaven Kinderklinik, Bruchweiler Edelsteinklinik Kinder-Reha, Böblingen Kinderklinik, Castrop-Rauxel Evangelisches Krankenhaus, Celle Klinik für Kinder- und Jugendmedizin, Chemnitz Kinderklinik, Chemnitz-Hartmannsdorf Innere Medizin - DIAKOMED-1, Coburg Innere Medizin, Coburg Kinderklinik, Coesfeld Kinderklinik, Coesfeld/Dülmen Innere Med., Darmstadt Innere Medizin, Darmstadt Kinderklinik Prinz. Margaret, Datteln Vestische Kinderklinik, Deggendorf Gemeinschaftspraxis, Deggendorf Medizinische Klinik II, Deggendorf Pädiatrie-Praxis, Delmenhorst Kinderklinik, Dessau Kinderklinik, Detmold Kinderklinik, Dinslaken Kinderklinik, Dornbirn Innere Medizin, Dornbirn Kinderklinik, Dortmund Kinderklinik, Dortmund Medizinische Kliniken Nord, Dortmund-Hombruch Marienhospital, Dortmund-St. Josefshospital Innere, Dortmund-West Innere, Dresden Neustadt Kinderklinik, Dresden Uni-Kinderklinik, Duisburg Malteser Rhein-Ruhr St. Anna Innere, Duisburg Sana Kinderklinik, Duisburg-St.Johannes Helios, Düren-Birkesdorf Kinderklinik, Düsseldorf Uni-Kinderklinik, Erfurt Kinderklinik, Erlangen Uni-Kinderklinik, Essen Diabetes-Schwerpunktpraxis, Essen Elisabeth Kinderklinik, Essen Kinderarztpraxis, Essen Uni-Kinderklinik, Esslingen Klinik für Kinder und Jugendliche, Eutin Kinderklinik, Feldkirch Kinderklinik, Filderstadt Kinderklinik, Flensburg Diakonissen Kinderklinik, Forchheim Diabeteszentrum SPP, Frankenthal Kinderarztpraxis, Frankfurt Diabeteszentrum Rhein-Main-Erwachsenendiabetologie (Bürgerhospital), Frankfurt Diabeteszentrum Rhein-Main-pädiat. Diabetologie (Clementine-Hospital), Frankfurt Uni-Kinderklinik, Frankfurt Uni-Klinik Innere, Frankfurt Uni-Klinik Innere2, Freiburg Kinder-MVZ, Freiburg St. Josef Kinderklinik, Freiburg Uni Innere, Freiburg Uni-Kinderklinik, Freudenstadt Kinderklinik, Friedberg Innere Klinik, Fulda Kinderklinik, Fürth Kinderklinik, Gaissach Fachklinik der Deutschen Rentenversicherung Bayern Süd, Garmisch-Partenkirchen Kinderklinik, Geislingen Klinik Helfenstein Innere, Gelnhausen Kinderklinik, Gelsenkirchen Kinderklinik Marienhospital, Gera Kinderklinik, Gießen Ev. Krankenhaus Mittelhessen, Gießen Uni-Kinderklinik, Graz Uni Innere, Graz Uni-Kinderklinik, Greifswald Uni-Kinderklinik, Gummersbach Oberbergklinikum, Göppingen Kinderklinik am Eichert, Görlitz Städtische Kinderklinik, Göttingen Uni-Kinderklinik, Hachenburg Kinderpraxis, Hagen Kinderklinik, Halberstadt Kinderklinik AMEOS, Halle Uni-Kinderklinik, Hamburg Altonaer Kinderklinik, Hamburg Endokrinologikum, Hamburg Kinderklinik Wilhelmstift, Hamburg-Nord Kinder-MVZ, Hameln Kinderklinik, Hamm Kinderklinik, Hanau Kinderklinik, Hanau diabetol. Schwerpunktpraxis, Hannover Kinderklinik MHH, Hannover Kinderklinik auf der Bult, Haren Kinderarztpraxis, Heide Kinderklinik, Heidelberg St. Josefskrankenhaus, Heidelberg Uni-Kinderklinik, Heidenheim Kinderklinik, Heilbronn Innere Klinik, Heilbronn Kinderklinik, Herdecke Kinderarztpraxis, Herdecke Kinderklinik, Herford Kinderarztpraxis, Herford Klinikum Kinder & Jugendliche, Heringsdorf Inselklinik, Herne Evan. Krankenhaus Innere, Herten St. Elisabeth Innere Medizin, Hildesheim Bernward Krks Kinderheilkunde, Hildesheim GmbH - Innere, Hildesheim Kinderarztpraxis, Hildesheim Kinderklinik, Hof Kinderklinik, Hohenmölsen Diabeteszentrum, Homburg Uni-Kinderklinik Saarland, Innsbruck Uni-Kinderklinik, Iserlohn Innere Medizin, Itzehoe Kinderklinik, Jena Kinderarztpraxis, Jena Uni-Kinderklinik, Kaiserslautern Kinderarztpraxis, Kaiserslautern-Westpfalzklinikum Kinderklinik, Kamen Klinikum Westfalen Hellmig Krankenhaus, Karlsburg Klinik für Diabetes & Stoffwechsel, Karlsruhe Städtische Kinderklinik, Kassel Klinikum Kinder- und Jugendmedizin, Kaufbeuren Kinderklinik, Kempten Oberallgäu Kinderklinik, Kiel Städtische Kinderklinik, Kiel Universitäts-Kinderklinik, Kirchen DRK Krankenhaus Kinderklinik, Kirchheim-Nürtingen Innere, Kleve Innere Medizin, Koblenz Kemperhof 1. Med. Klinik, Koblenz Kinderklinik Kemperhof, Konstanz Kinderklinik, Krefeld Innere Klinik, Krefeld Kinderklinik, Kreischa-Zscheckwitz Klinik Bavaria, Köln Kinderklinik Amsterdamerstrasse, Köln Uni-Kinderklinik, Landau Innere, Landshut Kinderklink, Lappersdorf Kinderarztpraxis, Leer Klinikum - Klinik Kinder & Jugendmedizin, Leipzig Uni-Kinderklinik, Leoben LKH Kinderklinik, Leverkusen Kinderklinik, Lienz BKH Kinderklinik, Lienz Diabetesschwerpunktpraxis für Kinder und Jugendliche, Limburg Innere Medizin, Lingen Kinderklinik St. Bonifatius, Linz KUK MedCampus IV Kinderklinik, Linz Krankenhaus Barmherzige Schwestern Kardiologie Abt. Int. II, Linz Krankenhaus der Barmherzigen Schwestern Kinderklinik, Lippstadt Evangelische Kinderklinik, Ludwigsburg Kinderklinik, Ludwigshafen Kinderklinik St.Anna-Stift, Ludwigshafen diabetol. SPP, Luxembourg - Centre Hospitalier, Lübeck Uni-Kinderklinik, Lüdenscheid Märkische Kliniken - Kinder & Jugendmedizin, Magdeburg Ki-Klinik St. Marienstift, Magdeburg Städtisches Klinikum Innere, Magdeburg Uni-Kinderklinik, Mainz Uni-Kinderklinik, Mannheim Uni-Kinderklinik, Marburg Uni-Kinderklinik, Marktredwitz Innere Medizin, Mechernich Kinderklinik, Meissen Kinderklinik Elblandklinikum, Memmingen Internistische Praxis, Memmingen Kinderklinik, Minden Kinderklinik, Moers Kinderklinik, Murnau am Staffelsee - diabetol. SPP, Mutterstadt Kinderarztpraxis, Mödling Kinderklinik, Mönchengladbach Kinderklinik Rheydt Elisabethkrankenhaus, Mühldorf am Inn Kinderarztpraxis, München 3. Orden Kinderklinik, München Diabetes-Zentrum Süd, München Kinderarztpraxis diabet. SPP, München Praxiszentrum Saarstrasse, München von Haunersche Kinderklinik, München-Gauting Kinderarztzentrum, München-Harlaching Kinderklinik, München-Schwabing Kinderklinik, Münster Herz Jesu Innere, Münster Ludgerus-Kliniken GmbH, Münster St. Franziskus Kinderklinik, Münster Uni-Kinderklinik, Münster pädiat. Schwerpunktpraxis, Münsterlingen Kinderklinik, Nauen Havellandklinik, Neuburg Kinderklinik, Neumarkt Innere, Neunkirchen Gemeinschaftspraxis Kinderheilkunde, Neunkirchen Marienhausklinik Kohlhof Kinderklinik, Neuruppin Kinderklinik, Neuss Lukas-Krankenhaus Kinderklinik, Neuss Lukaskrankenhaus Kinderklinik, Neuwied Kinderklinik Elisabeth, Neuwied Marienhaus Klinikum St. Elisabeth Innere, Nürnberg Cnopfsche Kinderklinik, Nürnberg Zentrum f Neugeb./Kinder & Jugendl., Oberhausen Kinderklinik, Oberhausen Kinderpraxis, Oberhausen St.Clemens Hospitale Sterkrade, Oberndorf Gastroenterologische Praxis Schwerpunkt Diabetologie, Oberwart - Burgenländische Krankenanstalten Pädiatrie, Offenbach/Main Innere Medizin, Offenbach/Main Kinderklinik, Offenburg Kinderklinik, Oldenburg Kinderklinik, Oldenburg Schwerpunktpraxis Pädiatrie, Olpe pädiatrische Gemeinschaftspraxis, Osnabrück Christliches Kinderhospital, Paderborn St. Vincenz Kinderklinik, Papenburg Marienkrankenhaus Kinderklinik, Passau Kinderklinik, Pforzheim Kinderklinik, Pfullendorf Innere Medizin, Pirmasens Städtisches Krankenhaus Innere, Plauen Vogtlandklinikum, Ravensburg Kinderklink St. Nikolaus, Regensburg Kinderklinik St. Hedwig, Rendsburg Kinderklinik, Reutlingen Kinderarztpraxis, Reutlingen Kinderklinik, Reutlingen Klinikum Steinenberg Innere, Reutte Tirol BKH Kinderklinik, Rheine Mathiasspital Kinderklinik, Ried Innkreis Barmherzige Schwestern, Rosenheim Innere Medizin, Rosenheim Kinderklinik, Rosenheim Schwerpunktpraxis, Rostock Uni-Kinderklinik, Rotenburg/Wümme Agaplesion Diakonieklinikum Kinderabteilung, Rüsselsheim Kinderklinik, Rüsselsheim MVZ, Saaldorf-Surheim Diabetespraxis, Saalfeld Thüringenklinik Kinderklinik, Saarbrücken Kinderklinik Winterberg, Saarlouis Kinderklinik, Salzburg Universitäts-Kinderklinik, Scheidegg Prinzregent Luitpold, Schw. Gmünd Stauferklinik Kinderklinik, Schweinfurt Kinderklinik, Schwerin Innere Medizin, Schwerin Kinderklinik, Schwäbisch Hall Diakonie Kinderklinik, Siegen Kinderklinik, Singen - Hegauklinik Kinderklinik, Singen Kinderarztpraxis, Spaichingen Innere, Speyer Diakonissen Stiftungskrankenhaus Pädiatrie, St. Augustin Kinderklinik, St. Johann Tirol Kinderklinik, St. Pölten Universitäts-Kinderklinik, Stade Kinderklinik, Stolberg Kinderklinik, Stuttgart Olgahospital Kinderklinik, Suhl Kinderklinik, Sylt Rehaklinik, Tettnang Innere Medizin, Traunstein Kinderklinik, Traunstein diabetol. Schwerpunktpraxis, Trier Kinderklinik der Borromäerinnen, Trostberg Innere, Tübingen Uni-Kinderklinik, Ulm Endokrinologikum, Ulm Endokrinologikum Amedes, Ulm Schwerpunktpraxis Bahnhofsplatz, Ulm Uni-Kinderklinik, Vechta Kinderklinik, Viersen Kinderkrankenhaus St. Nikolaus, Villach Kinderklinik, Villingen-Schwenningen Schwarzwald Baar Klinikum Kinderklinik, Villingen-Schwenningen Schwarzwald-Baar-Klinikum Innere, Vöcklabruck Kinderklinik, Waldshut Kinderpraxis, Waldshut-Tiengen Kinderpraxis Biberbau, Wangen Oberschwabenklinik Innere Medizin, Waren-Müritz Kinderklinik, Weiden Kinderklinik, Weingarten Kinderarztpraxis, Weisswasser Kreiskrankenhaus, Wels Klinikum Pädiatrie, Wernberg-Köblitz SPP, Werningerode MVZ, Wesel Marienhospital Kinderklinik, Wien 3. Med. Hietzing Innere, Wien Preyersches Kinderspital, Wien Rudolfstiftung, Wien SMZ Ost Donauspital, Wien Uni-Kinderklinik, Wien Wilhelminenspital 5. Med. Abteilung, Wiesbaden Helios Horst-Schmidt-Kinderkliniken, Wiesbaden Kinderklinik DKD, Wilhelmshaven Kinderarztpraxis, Wilhelmshaven Klinikum Kinderklinik, Winnenden Rems-Murr Kinderklinik, Wismar Kinderklinik, Wittenberg Innere Medizin, Wittenberg Kinderklinik, Wolgast Innere Medizin, Worms - Weierhof, Worms Kinderklinik, Wuppertal Kinderklinik, Würzburg Kinderarztpraxis, Zweibrücken Kinderarztpraxis, Zwettl Landesklinikum Gmünd-Waidhofen Kinderklinik,
